# Strategies to reconstruct 3D *Coffea arabica* L. plant structure

**DOI:** 10.1186/s40064-016-3762-4

**Published:** 2016-12-05

**Authors:** Fabio Takeshi Matsunaga, Jonas Barbosa Tosti, Armando Androcioli-Filho, Jacques Duílio Brancher, Evelyne Costes, Miroslava Rakocevic

**Affiliations:** 1 UEL, Rodovia Celso Garcia Cid, Pr 445 km 380, P.O.Box 10011, Londrina, PR 86057-970 Brazil; 2 IAPAR, Rodovia Celso Garcia Cid, km 375, P.O.Box 481, Londrina, PR 86047-902 Brazil; 3 UMR AGAP Equipe Architecture et Fonctionnement des Espèces Fruitières, INRA, Avenue Agropolis, TA A-96/03, 34398 Montpellier, France; 4 Institute of Biology, UNICAMP, Rua Monteiro Lobato, 255 - Cidade Universitária, Campinas, SP 13083-862 Brazil; 5 Embrapa Informática Agropecuária, Avenida André Tosello 209, P.O.Box 6041, Campinas, SP 13083-886 Brazil

**Keywords:** Plant architecture, Berry distribution, Metamer, LAI, Vertical profile, Gaussian model, Python

## Abstract

Accurate model of structural elements is necessary to model the foliage and fruit distributions in cultivated plants, both of them being key parameters for yield prediction. However, the level of details in architectural data collection could vary, simplifying the data collection when plants get older and because of the high time cost required. In the present study, we aimed at reconstructing and analyzing plant structure, berry distributions and yield in *Coffea arabica* (Arabica coffee), by using both detailed or partial morphological information and probabilistic functions. Different datasets of coffee plant architectures were available with different levels of detail depending on the tree age. Three scales of decomposition—plant, axes and metamers were used reconstruct the plant architectures. CoffePlant3D, a software which integrates a series of mathematical, computational and statistical methods organized in three newly developed modules, AmostraCafe3D, VirtualCafe3D and Cafe3D, was developed to accurately reconstruct coffee plants in 3D, whatever the level of details available. The number of metamers of the 2nd order axes was shown to be linearly proportional to that of the orthotropic trunk, and the number of berries per metamer was modeled as a Gaussian function within a specific zone along the plagiotropic axes. This ratio of metamer emission rhythm between the orthotropic trunk and plagiotropic axes represents the pillar of botanical events in the *C*. *arabica* development and was central in our modeling approach, especially to reconstruct missing data. The methodology proposed for reconstructing coffee plants under the CoffePlant3D was satisfactorily validated across dataset available and could be performed for any other Arabica coffee variety.

## Background

The coffee tree architecture, described as Roux’s model, is a characterized by a continuous growth and dimorphic axes (Hallé et al. 1978). An orthotropic axis of 1st order forms, at each node, two syllepetic plagiotropic axes of 2nd order, even though sometimes, no branch, or just one develops. The orthotropic axis respects an opposite-decussate phyllotaxy. The lateral axes follow an orthogonal—decussate pattern of leaf initiation of orthotropic axis, but both internode torsion and petiole angle reorient leaves, resulting in dorsiventral phyllotaxy (Dengler 1999). In *Coffea canefora*, the 2nd order axes are rarely branched in 3rd order ones, while in *Coffea arabica* L. (Arabica coffee), the plagiotropic axes develop from the 2nd to the 5th orders. The highest axes orders appear in three to four years after pruning (Rakocevic and Androcioli-Filho 2010).

*Coffea* *canefora* has been the first vegetative species to be modeled based on stochastic processes for representing growth distribution and branching. And in early stages, linear regression linking the number of metamers emitted on 1st and 2nd order axes is defined (de Reffye 1981). Despite numerous studies and models previously developed on *C*. *canefora* (Cilas et al. 2006) no model presently exists for *C*. *arabica*. Such model would allow predicting 3D tree architecture, berry distribution within tree structure and yield over time. For modeling the foliage distribution, berry distribution and yield, an accurate model of structural elements dynamics is thus necessary for this species. A first module called ‘VirtualCafe3D’ (Matsunaga and Rakocevic 2011) was previously developed to reconstruct coffee mock-ups from local geometrical and topological measurements. This development mainly allowed us to overcome some limitations in VPlants (Pradal et al. 2009), which referred to branch and leaf dimorphism observed in *Coffea* sp. In ‘VirtualCafe3D’, geometrical adjustments of branch cardinal orientation and spatial distribution of the two leaves in a pair at a node were achieved by insertion of very short “virtual” internodes.

Plant architecture can be described at various degrees of detail (Barthélémy 1991; Godin et al. 1999a), from very detailed description, when all plant metamers are measured, to low detailed description, when only some of components are measured. Intermediate and mixed levels of details can also be considered, when parts of the plants are described with low details while others are described with high detailed measurements (Godin 2000). Whatever is the detail level, data for plant 3D reconstructions could be collected by diverse methods of digitizing (Godin et al. 1999a; Li et al. 2015; Preuksakarn 2012) or local measurements (Godin et al. 1999a; Rakocevic and Androcioli-Filho 2010). In experiments, we carried out over years, architectures of Arabica coffee plants were collected with different levels of details and different sampling strategies, depending on the plant age and observation year. This generated a general problem of 3D reconstruction of plants with missing data, to fully benefit from the data acquired. To face this problem, a second module—‘AmostraCafe3D’ was proposed (Rakocevic et al. 2013; Matsunaga et al. 2014). In this module, the berry presence and number were modeled by a Gaussian function, considering as parameters the maximum number of berries and the interval of metamer ranks at which berry may occur along 2nd order axes. Even though such Gaussian functions were able to reproduce the asymmetry of the maximum number of berries in a specific zone of berry appearance along 2nd order axes, the estimation of mean berry number per 2nd order axes were under-estimated.

In the present study, we aimed at further improving our strategy to be able to reconstruct a 3D structure of whole *C*. *arabica* trees using either detailed or partial morphological information collected on whole plants, as branching structure and leaf/berry distribution. For this purpose, several modules for reconstruction of virtual plants were integrated to make the ‘CoffePlant3D’ system. Following previous findings on we hypothesized that the growth of 2nd order axes could be linearized with respect to that of the orthotropic trunk. The berry distribution was hypothesized to follow a Gaussian function with berries number mean at boundaries of zones of appearance along the orthotropic and plagiotropic axes. With these new assumptions we built CoffePlant3D system.

## Results

### Software development

**Fig. 1:**
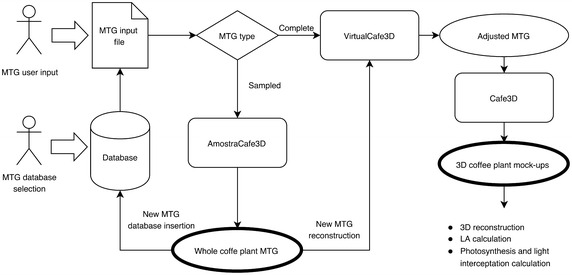
CoffeePlant3D dataflow linking complete or partially detailed MTG of coffee plants, MTG database, AmostraCafe3D (inclusion of missing data at metamer scale), VirtualCafe3D (geometrical correction of axes orientation and leaf pair position and orientation) and Cafe3D (visualization of the 3D plant structure) modules to final 3D coffee mock-ups

CoffePlant3D software, dedicated to 3D coffee reconstructions, integrated three interconnected modules (AmostraCafe3D, VirtualCafe3D and Cafe3D) and processes, ranging from user interface to input of information related to plant, field and productivity (Fig. 1). All modules of CoffeePlant3D were developed with Python language (Borcherds 2007) under PyCharm IDE, using NumPy and SciPy libraries and packages for numerical and statistical methods, respectively. AMLPy was used for multiscale tree graph (MTG) architecture manipulation, and PlantGLViewer (Pradal et al. 2009) for plant geometric reconstruction and visualization.

In CoffePlant3D, the output of one module was the input to the next one performing an automatic process flow (Fig. 1). The initial input of CoffeePlant3D can be a MTG file created by the user or selected from the database as a MTG like conditions of interest (year of production—PY, spatial plant arrangement). The datasets and extracted data were stored in a database, projected in Oracle. The entity-relationship model (Codd 1970) was applied to represent the data storage, aiming at modeling the relationship between entities. Entities data were inserted, queried and updated by Structured Query Language scripts. The database was modeled in a normalized form (Codd 1982), optimizing the data representation and avoiding data redundancy.

The hierarchy of CoffePlant3D software was represented by multiple class inheritance and composition, using object-oriented programming concepts (Kindler and Krivy 2011). The data structure was composed by three main object classes, corresponding to the MTG scales. The recursive routing algorithm (Javanian and Vahidi-Asl 2006) was applied to convert the CoffeePlant3D data structure to MTG or vice versa. This algorithm routed all metamer objects, starting from the orthotropic axis, and writing all metamers respecting the hierarchy of branching orders. The temporal algorithm complexity was *O*(log(*n*)), where log(*n*) represents the average time processing in a tree node search, since the tree data structure is efficient to search elements and nodes.

### Reconstruction from partial or simplified datasets

When coffee plants were sampled under mixed levels of details, the MTG needed to be topologically complemented by AmostraCafe3D module. This module receives as input one MTG with mixed levels of details about axes and performs the reconstruction of missing data. For this, both partially and detailed measured 2nd order axes were classified by their position along the orthotropic axis, considering 40 cm-thick layers along the vertical tree profile (Rakocevic et al. 2013). The total numbers of berries and axis length were used as variables to classify all axes (partially and completely described) that appeared on the same cardinal orientation, using the *k*-means method. In our case, two or three classes and two to five vertical layers were considered, depending on the tree height.

The AmostraCafe3D considered four attributes for each metamer in reconstruction: leaf, branching and berry presence or absence and internode length. Their probabilities were generated based on properties of completely measured axes classified in the same cluster, attributing the presence or absence at each node along the partially measured 2nd order axes. These probabilities were randomly chosen by running the ‘choice’ function, which uses the Mersenne twister as the core generator (Katzgraber 2010). The input of this function was a binary vector *Vi* of *n* positions (for leaf/branching/berry presence), and it returns one random element from this vector as output, respecting the generated probability. The Mersenne twister generates pseudorandom number generator (PRNG), for generating a sequence of numbers *x*_*1*_, *x*_*2*_, …, *x*_n_ using a recurrence defined as $x_{i} = f\left( {x_{i - 1} ,x_{i - 2} , \ldots ,x_{i - n} } \right)$, where *n* is the initial numbers needed to start the recurrence. All generated PRNGs in AmostraCafe3D produced the binary vector *V*_*i*_ of 0 and 1. This procedure was recursively applied to 3rd 4th and 5th order axes, when the vectors representing the berry presence or absence were generated. A different approach was performed for the leaf and branching presence or absence, where the whole structure of 3rd, 4th and 5th order axes was copied from completely measured to partially measured axes, but respecting the synchronization of events. The reconstruction of each internode length, along 2nd order partially measured axes, was based on point wise estimated probability of real length and average length of the internodes obtained from the completely described axes situated in the same cluster and ‘choice’ function.

It was assumed that the second order axes have the same phyllochron with 3rd, 4th and 5th order axes. A linear model was applied, permitting the synchronization to the emitted metamer number in partially measured axes. The rule applied to generate the correct index at each metamer was defined as follows: $y = (a - b) + x,\;if\;a > b;\;y = x - (b - a),$ $if\;a < b\;and\;x > (b - a);$ $y = 1,$ $if\;x < (b - a)$ where the variables *a* and *b* represented the orthotropic rank axis to be reconstructed and the orthotropic rank of the complete measured axis, respectively. The variable *x* represents the metamer index being reconstructed, while *y* represents the metamer index on the measured axis equivalent to *x*.

After clustering the branches by *k*-means method, the partially measured axes reconstruction started, respecting the indexes from the bottom to the top. The length and number of metamers of the 2nd order axes, as well as the zones containing mature (*mb*) and immature berries (*ib*), were estimated depending on their rank along the orthotropic axes and field treatments.

### Axes growth depending of year of production and cultural practices

**Fig. 2:**
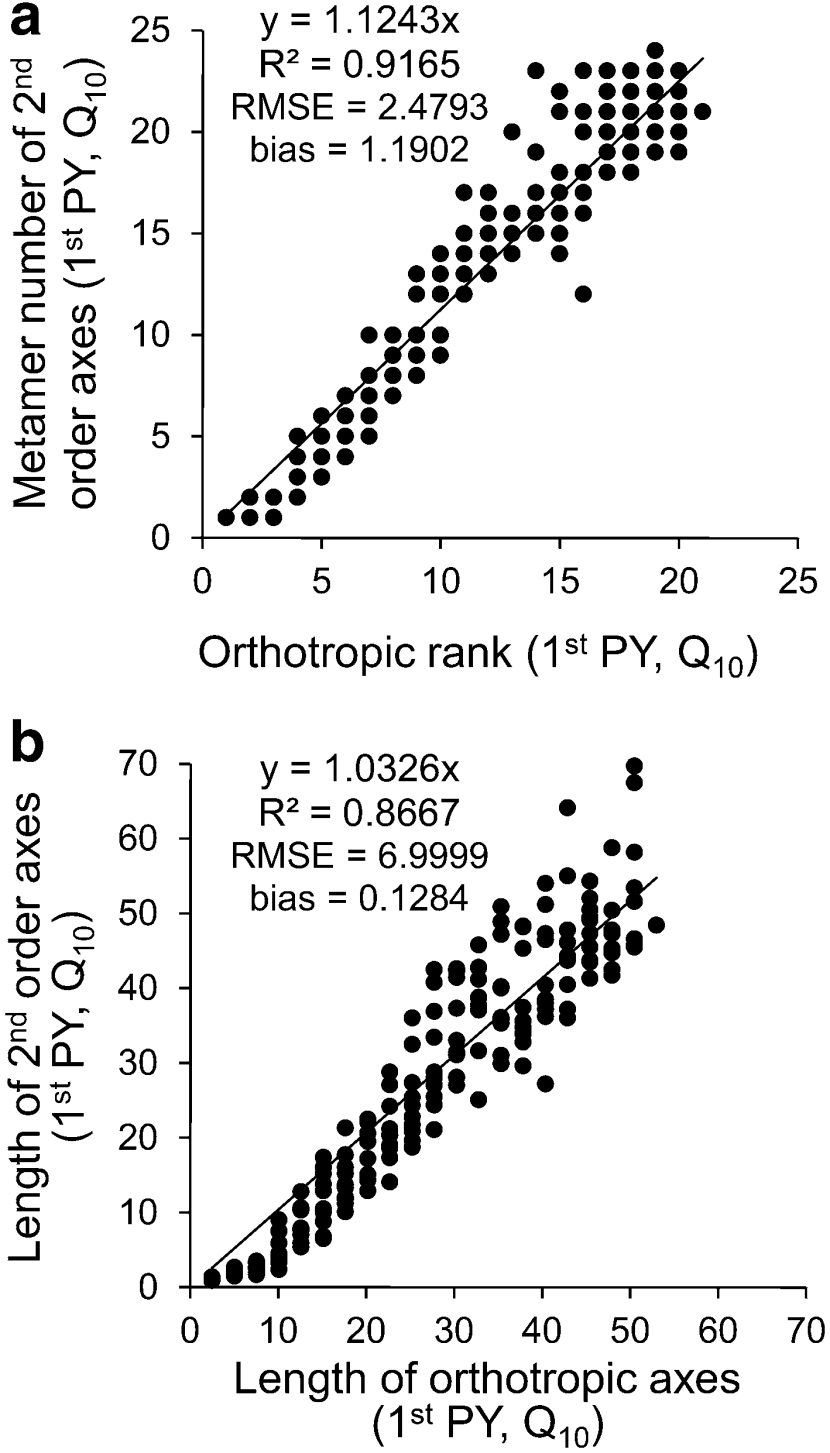
Linear model established in the 1st production year between: **a** measured number of metamers of 2nd order axes and their rank of insertion on orthotropic trunk and **b** measured length of 2nd order axes and the length above their insertion on orthotropic trunk. Example of square planting pattern and 10,000 plants per ha^−1^—Q_10_ dataset

**Fig. 3:**
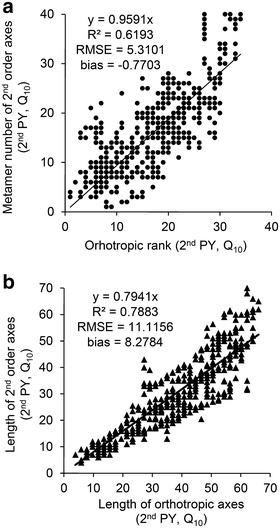
Linear model established in the 2nd production year between: **a** measured and computed number of metamers of 2nd order axes and their rank of insertion on orthotropic trunk and **b** measured length of 2nd order axes and the length above their insertion on orthotropic trunk. Example of square planting pattern and 10,000 plants per ha^−1^—Q_10_ dataset

The mean number of metamers and length of the 2nd order axes were linearly dependent on their insertion rank from the top of the trunk (Table 1; Figs. 2, 3). In the 1st PY, the regression coefficient between the number of metamers of the 2nd order axes and the number of metamers of the orthotropic axis above their insertion rank was close to 1 (Table 1). Its values decreased with tree development and years, from more than 1 to 0.91 in Q_10_ and from 0.97 to 0.71 in R_6_. They also varied between cultural practices, with lower values observed for R PP (0.87 and 0.97 for R_10_ and R_6_ in 1st PY, respectively) than Q PP (1.12 and 1.00 for Q_10_ and Q_6_ in 1st PY, respectively; Table 1). This means that the 2nd order axes emitted slightly less metamers when the plant was grown in R PP. When the length of 2nd order axes was related to the same variable on the orthotropic axes, measured above the insertion rank, higher slopes were observed in 1st than 2nd PY (Table 1; Figs. 2, 3). Obviously, the number of metamers increased with years in both 1st and 2nd order axes (Fig. 2a vs. Fig. 3a).

**Table 1:** Coefficient of the linear regressions between the number of metamers and the length of orthotropic and plagiotropic axes

PY	Variable	Q_10_	Q_6_	R_10_	R_6_
1st	Metamer number	1.124	1.000	0.871	0.974
	Length of 2nd order axes	1.033	0.891	0.828	0.960
2nd	Metamer number	0.959	1.138	0.895	0.930
	Length of 2nd order axes	0.794	0.859	0.931	0.798
6th	Metamer number	0.956	0.868	0.887	0.793
7th	Metamer number	0.905	0.930	0.894	0.705

Plants grown under two densities (6000 and 10,000 plants ha^−1^) and planting patterns—PP (square—Q and rectangular—R) in 1st, 2nd, 6th and 7th production year (PY) after the pruning

The 2nd order axes were filled by metamer number based on the length probability of each internode and the defined regression coefficients (Table 1) plus their respective R^2^, all stored in the database. The regression coefficients had a role to adjust the metamer number to the total measured axis length based on the predicted length and to consider a range of variability consistent with R^2^. Finally, the measured axes length was attributed. The difference between the expected and measured axes was either added or subtracted to the length of each internode.

### Computing the berry distribution

The next step was to compute the berry distribution based on metamer number, vectors of berry presence/absence and measured total berry number per axes. The equations for berry distribution were obtained by manipulation of experimental datasets extracted from MTGs, which were crossed and combined to logical rules referent to the Arabica coffee architectural patterns. Incidence range (initial and final index of berry occurrence) and the respective berry number on the boundaries, maximum productivity value and their position index considering all branching orders, were used in Gaussian function to model berry distribution along the 2nd order axes: $f(x) = G(x) + \left( {N_{b} (i) - G(i)} \right);$ $G(x) = a\,{ \cdot } \exp \left( { - \left( {x - b} \right)^{2} /\left( {2c^{2} } \right)} \right)$ where *a* was the curve height, defined as the maximum number of berries (*mb* or *ib*); *b* was the position of a curve peak center, defined as the local of highest incidence of berries; *c* the controlled length of the sinus; *x* was the index of a 2nd order axes internode; *i* was the region (limits determining the region of berries incidence, i.e. the beginning and the end) of the respective value of berry number *N*_*b*_(*i*). If *x* < *a*, *i and N*_*b*_(*i*) considered the beginning, while if *x* > *a*, *i and N*_*b*_(*i*) considered the end of the region. The modification of curve center allowed the curve displacement, while the values of extreme limits allowed the deformation and asymmetry of the curve. After the *Vi* and Gaussian function definition, the total berry number was distributed proportionally over the axes metamers, considering the proportional values attribution over the metamer rank with berry existence (1 on the *V*_*i*_): $B(i) = {\text{Ceil}}\left( {(N_{b}{ \cdot } V_{i} )/{\sum }V_{i} } \right),$ for 0 ≤ *i* ≤ *n*, where *B*(*i*) is the computed berry number (for *mb* and *ib*) to be attributed in a metamer *i*, *N*_*b*_ is the total berry number obtained from MTG, *n* is the number of the metamers and the Ceil(*x*) function was applied to round the berry number up to attribute the non-zero values, when the *V*_*i*_ value is equals to 1. The same methodology was applied on 3rd, 4th and 5th order axes.

### Accuracy of axes and berry distributions on trees reconstructed with AmostraCafe3D

The accuracy of axis attributes (i.e. number of metamers, length and number of berries) obtained with AmostraCafe3D was verified in the 2nd PY, in which low detailed measurements were performed, by comparison with the 1st PY in which the metamer number was measured on all axes. The dispersion in the estimated number of metamers of the 2nd order axes, depending on their rank along orthotropic axes, increased in 2nd PY (Fig. 3a) compared to the 1st PY (Fig. 2a), as shown by comparing the R^2^ (0.62 vs. 0.92), RMSE (5.31 vs. 2.48) and, to a lesser extent, bias values (−0.77 vs. 1.19 at Fig. 3a vs. Fig. 2a). Similar observation could be done about the 2nd order axes length estimations in 2nd PY (Fig. 3b) compared to length measurements in the 1st PY (Fig. 2b), with R^2^ (0.79 vs. 0.87), RMSE (11.12 vs. 6.99) and bias (8.28 vs. 0.13), respectively. However, we decided that these values of R^2^, RMSE and bias were acceptable for modeling of metamer and length distribution at axis scale to further proceed to validation at more integrated scales.

**Fig. 4:**
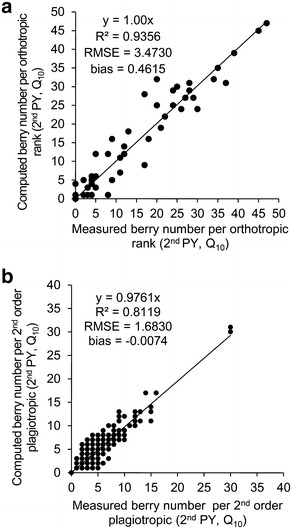
Validation of the Gaussian model for berry distribution. Comparison between measured and computed number of berries: **a** on 2nd order axes inserted along orthotropic trunk and **b** along ranks of 2nd order axes. Example of square planting pattern and 10,000 plants per ha^−1^—Q_10_ dataset in the 2nd production year

The measured and computed sum of berries of the 2nd order axes were compared using the whole dataset regardless the particular square planting pattern under 10,000 plants per ha^−1^ (Q_10_) in the 2nd PY (Fig. 4). The berry occurrence was observed across a vertical profile, i.e. on the 2nd order axes inserted along the orthotropic trunk (Fig. 4a) and across the horizontal profile, i.e. along the 2nd order plagiotropic axes (Fig. 4b). The Gaussian model of berry distribution that was used to estimate berry distributions was considered accurate, considering the R^2^ close to 1 (1.00 and 0.98, along the vertical and horizontal profiles, respectively).

### Visualization of the mock-ups

The software flow (Fig. 1) proceeded to reconstruction and visualization module (Cafe3D). All vertices of the MTG, considering the three scales of decomposition were extracted, and a ‘Dressing Data’ file from the PlantFrame function—an AMLPy method (Godin et al. 1999b; Pradal et al. 2009) was used to define the geometrical structure of the coffee plant. For berries, an ellipsoidal form was attributed, simpler than the complex geometrical fruit modeling (Tinoco et al. 2014).

The general data structure permitted the lecture, conversion, creation and further processing of MTG that represented coffee plants. Depending on the detail degree of the input (mixed or high detailed), the processing might not use all the modules for the 3D reconstruction. If the leaf length and width were defined, the reproduction of individual 3D coffee leaf, constructed on 16 triangles, was performed by reduction of leaf length and width equally to the measured values of individual leaf surface (Rakocevic et al. 2013). The functions applied were: length = *len* × √0.685 and width = *wid* × √0.685.

**Fig. 5:**
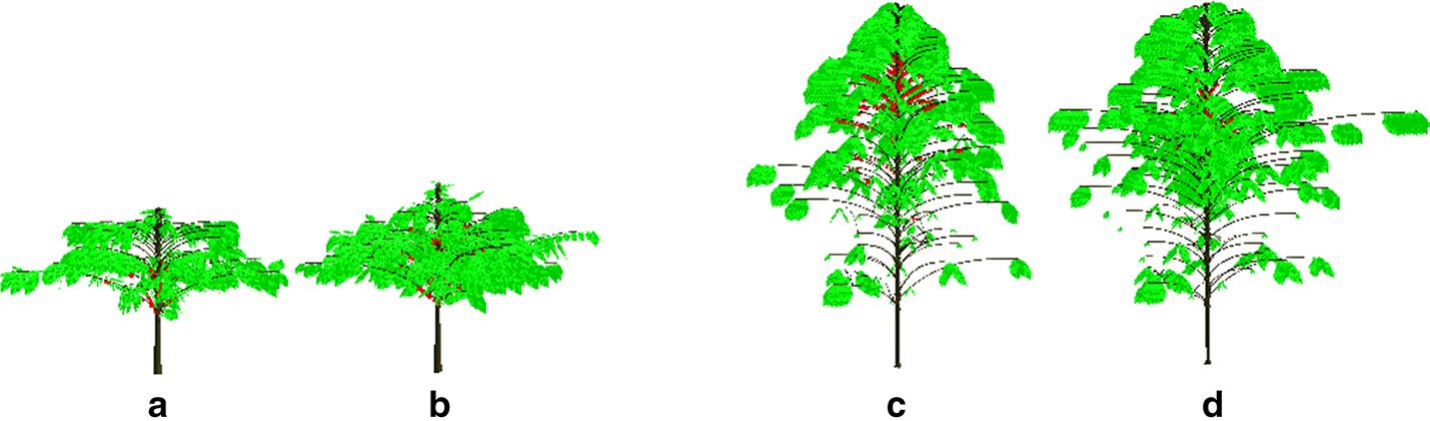
Mock-ups of average Arabica coffee plants in: **a** 1st, **b** 2nd, **c** 6th and **d** 7th production years. Example of square planting pattern and 10,000 plants per ha^−1^—Q_10_ dataset

The mock-ups were considered the final output of CoffePlant3D software. The visualization of average coffee plant reconstructed for four observed production years, i.e. 1st, 2nd, 6th and 7th PY cultivated under Q_10_ planting design, was performed considering the periods of berry ripening (Fig. 5).

### Accuracy of the leaf area (LA) and mock-ups reconstructed with AmostraCafe3D at plant scale

**Fig. 6:**
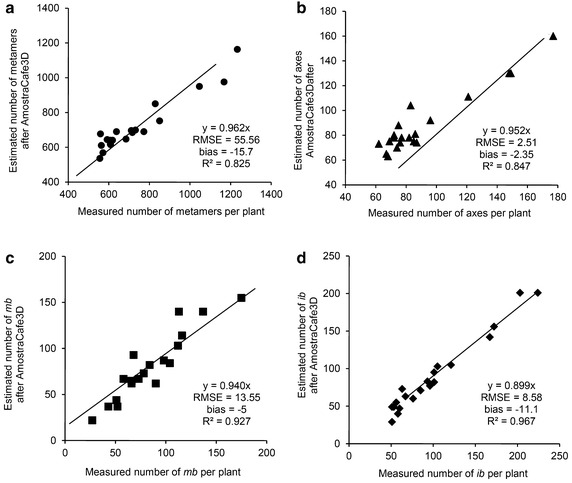
Comparison between measured structural and reproductive parameters, based on detailed dataset and estimated after AmostraCafe3D processing on the reduced dataset: **a** number of metamers per plant, **b** number of axes per plant, **c** number of mature berries (*mb*) per plant and **d** number of immature berries (*ib*) per plant

The reduced dataset was used to test the adequacy of reconstructions with AmostraCafe3D at the plant scale, including the total number of metamers (Fig. 6a), axes (Fig. 6b), *mb* (Fig. 6c) and *ib* (Fig. 6d) per plant. The dispersion in estimations of the four variables was acceptable according to R^2^ (0.83, 0.85, 0.93 and 0.97) and RMSE compared to mean value (55.56–733.15, 2.51–91.3, 13.55–93.35 and 8.58–10.15), respectively (Fig. 6a–d). The average bias indicated a general underestimation of all estimated variables: −15.7 for the total number of metamers per plant (Fig. 6a) and −2.35 for the total number of axes per plant (Fig. 6b), likely to result from rules used to reconstruct high order axes (3rd order axes appeared in the 1st PY). The berry number was reconstructed with high accuracy, with the bias of −5 and −11.1, for the total *mb* and *ib* per plant (Fig. 6c, d, respectively).

**Fig. 7:**
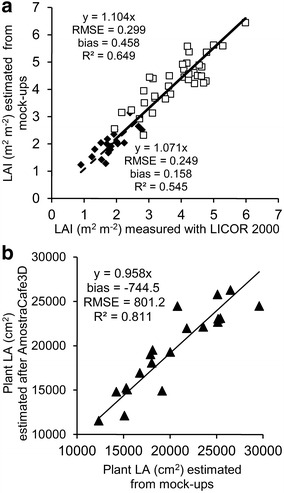
Validation of the leaf area parameters at plant scale: **a** measured and estimated LAI from mock-ups in the 1st and 2nd PY and **b** LA of plants estimated from mock-ups based on detailed dataset and obtained after AmostraCafe3D processing of the reduced dataset

The leaf area index (LAI) of reconstructed mock-ups, based on either detailed or partial datasets was estimated under the VegeSTAR for the 1st PY and the 2nd PY and compared to the measured values obtained with LICOR-2000 (Fig. 7a). The reconstructed LAI was accurate, considering the average bias and RMSE values (0.158 and 0.249 for the 1st PY, and 0.458 and 0.299 for the 2nd PY, respectively). The bias values suggested that the LAI estimated from the mock-ups were in average slightly overestimated compared to measured values. The LA issued from the detailed description of twenty plants collected 1st PY was compared with the same data after its reduction to a partial dataset (Fig. 7b). The reduced dataset was posteriorly processed with AmostraCafe3D to be modeled into the three scales described, and fitted to the original data. The RMSE for LA was low (801.2 cm^2^ compared to mean value 20,271 cm^2^). The LA estimation after AmostraCafe3D was slightly underestimated compared to the original detailed datasets (average bias = −744.5 cm^2^).

## Discussion

In this study, we proposed an empirical approach to model the 3D structure and berry distribution of *C*. *arabica* based on experimental data collected manually. This led us to build static reconstructions of coffee plants at three scales and for several dates of berry ripening. The final CoffePlant3D out-puts were accurate mock-ups that could be used for further ecophysiological and environmental manipulation and calculation. Our strategy was close to methods based on a limited number of partial measurements of organs geometry on which allometric relationships are applied to reconstruct plant architecture from organ scale to the entire stand scale (e.g. Casella and Sinoquet 2003). Another strategy could have been to record 3D points by digitizing (Sinoquet et al. 1997), but this method has been shown to be also highly time consuming, and not adapted to describe many individuals. More recently, reconstruction methods based on image processing (Phattaralerphong and Sinoquet 2005; Quan et al. 2006), 3D laser scanner (Preuksakarn 2012), and 3D vision sensors (Nakarmi and Tang 2012) have been proposed and are likely to improve data collection efficiency. Image-based approaches proved useful in fast phenotyping providing the information needed to compute plant traits summary, such as total LA or mean leaf angle (White et al. 2012). However, many difficulties still should be overcome, regarding organ occlusions or accessibility in high-density planting systems, before data issuing from such alternative methods could be performed on coffee tree and integrated in the CoffeePlant3D pipeline we proposed herein.

The coffee plants reconstructed with CoffeePlant3D was shown to be accurate, especially in berry distribution, number of metamers and axes, and LA. At plant scale, LAI estimations were validated by the measurements performed with LICOR 2000 since the overestimation was about 10%, i.e. within a range considered acceptable for coffee plants (Angelocci et al. 2008). Our modeling approach was based on the exploration of rich databases and rules derived from coffee plant architecture. A central rule we used was based on the linear regression linking the number of metamers emitted on 1st and 2nd order axes. This growth pattern, herein observed in *C*. *arabica*, was similar to that previously defined between the orthotropic and 2nd order axes growth at early stages of *C*. *canefora* vegetative growth (de Reffye 1981). The emission rate was shown to decrease with plant age and depended on branching order in *C*. *arabica*, thus following classical ontogenetic rule previously observed by Dauzat et al. (2012).

In many species, the plant dynamics showed synchronous emergence of organs, as for instance in rice the synchronized emergence of leaves on the main stem and on the tillers up to flowering (Jaffuel and Dauzat 2005). A similar synchronization in the emergence of metamer number, depending on position and branching order was accounted in our computational approach for coffee mock-up reconstructions. In CoffePlant3D, the probability of ‘choice’ function was used to access the information from axes to metamers scales, if the included variables were independent (Magidson and Vermut Magidson and Vermunt 2002). Moreover, this function was fitted to the observed values of the number of metamers, berries and leaves in the whole plant. The intensity of berry distribution along plagiotropic axes was modeled by Gaussian function in CoffeePlant3D, considering the probability of berry occurrence within specific zones. The Gaussian function is a parametric and low-cost function appropriate for modeling biological and natural systems, as responses of wind plant turbulence (Wu and Infield 2013), real-life behavior framework for non-playable character simulation (Kyungeun et al. 2007), or for modeling the plants size variation in maize plots (Picoli et al. 2012). This strategy permitted the reconstructions of Arabica coffee plants at specific static stages of berry ripening.

To reconstruct Arabica coffee plant at any stage, further developments of CoffePlant3D will be required, for instance by exploring Markovian models and dynamic functions. Indeed, Markovian models (Costes and Guédon 2002; Renton et al. 2006), L-systems (Prusinkiewicz and Lindenmayer 1990; Loik and Cournède 2008), or dynamic functions—Poison law and binomial (de Reffye 1981), have been successfully applied in mechanistic and probabilistic models, such as GreenLab (Kang et al. 2007), MAppleT (Costes et al. 2008) and LPeach (Lopez et al. 2010) to generate dynamically 3D plant structures.

## Conclusions

The 2nd order axes growth was shown to be linearly correlated to that of the orthotropic axis, this synchronizing the branching structures and berries distribution in both the vertical and horizontal profiles. This dynamic is a pillar event in the Arabica coffee life and was central in our modeling approach at all scales, from plant to metamer scale. Reconstructions of coffee plants under the CoffePlant3D could now be performed for any other Arabica coffee variety in a period of production of plants. Our research efforts will focus in the next future on the development of a dynamic model of coffee tree growth, in interaction with its environment, including the apex mortality and variations on phyllochron depending on temperature and water availability.

## Methods

### Plant material and architectural data collection

The experiment was set up at the Agronomical Institute of Paraná (IAPAR), Londrina (23°18′S and 51°17′W, 563 m above the sea level), Brazil, with adult *C*. *arabica* trees, of the most popular Paraná cultivar—IAPAR 59. The seedlings were planted in 1995 and pruned close to the ground (1st in 2000 and 2nd in 2008). Two high plant densities (6000 and 10,000 plants ha^−1^) combined to two planting patterns (PP), square (Q) and rectangular (R) defined four treatments identified as Q_10_, R_10_ (3 m × 0.33 m), Q_6_ (1.29 m × 1.29 m) and R_6_ (3 m × 0.55 m).

The plants were coded as a Multi Scale Tree Graph—MTG (Godin and Caraglio 1998), under VPlants. The coffee plant topology was decomposed into three scales: the plant, the axes and the metamer scale in which two distinct subclasses were used to distinguish the axes they belong to, orthotropic and plagiotropic (Rakocevic and Androcioli-Filho 2010). The branching process started from the orthotropic axis (1st order) to higher axes orders (up to 5th), which were recursively defined.

The level of details in data collection could vary, simplifying the detailed collection when older coffee plants were measured, because of the high time cost for data collection. Whatever was the level of detail, the orthotropic axis was always described at the metamer scale. A very detailed architectural description was performed in the 1st PY after the low pruning, in June 2010, before the first berry collection. For each plagiotropic axis was defined the length of each internode, length/width/elevation angle/cardinal orientation of leaves, number of mature (*mb*) and immature berries (*ib*) and position/orientation/total length of borne plagiotropic axes.

When plants were more developed (2nd, 6th and 7th production year) partially detailed data collection was performed. Four 2nd order axes were sampled (each one oriented to one cardinal point), in each 40 cm-thick layer along a vertical tree profile. These axes were described at the metamer scale, as well as their lateral axes of 3rd to 5th order. The detailed description of plagiotropic metamers included the same parameters as for orthotropic metamers, plus *mb* and *ib* number. All other 2nd order axes were described by their position along the orthotropic trunks, total length, *mb* and *ib* number, elevation angle and cardinal orientation.

To validate the computational methods used for reconstructions of coffee plants, the very detailed dataset collected in 2010, the 1st production year (PY), was transformed into a partially detailed dataset. Four 2nd order axes, oriented to four cardinal points, were maintained in each 40 cm-thick layer with their detailed description at metamer scale. Data collection on all the other axes was reduced to their attributes, i.e. total length, elevation, total *mb* and *ib* number. The accuracy of 3D reconstruction obtained from the simplified dataset was tested for the total number of metamers per plant, number of axes per plant and leaf area index (LAI). This latter variable was estimated from mock-ups in VegeSTAR (Adam et al. 2006) and measured with LICOR 2000 in the 1st and 2nd PY, with an indirect method adapted for coffee plants on a set of ten shots: the first one was made above the canopy, the next eight below the plant crown at 5 cm and 35 cm from the trunk and oriented to four cardinal points; the tenth shot was again performed above the canopy. Measurements were performed on 20 plants in the 1st PY and on 36 in the 2nd PY.

Data extraction from the MTGs was performed using AMAPstudio—Xplo software (Griffon and de Coligny 2014). A special attention was paid on berry distribution along the orthotropic trunk and along 2nd to 5th order axes. The extracted data (number of axes metamers per axes, length of axes, number of berries per rank/metamer/axes) were used to generate relationships between the metamer number/length of 1st and 2nd order axes, as for the berry distribution. Those relationships, together with boundaries and zones of berry distribution were included into a database linked to CoffeePlant3D software.

### Statistical analyses

Statistical analyses were performed with R.3.2.2 (R Development Core Team 2015). Linear regressions were used to compare the estimations of measured plants versus those processed under CoffeePlant3D on the following variables: number of metamers along the orthotropic and plagiotropic axes, number of lateral axes. R^2^, RMSE and medium bias were used as indicators of the reconstruction quality.
